# Health Tract No. 326

**Published:** 1868-06

**Authors:** 


HEALTH TRACT No. 326.

### RE-VACCINATION.

Some years since, we suggested that a new supply of vaccine matter should be obtained from the cow, as that which was taken half a century ago, would necessarily loose its influence in protecting men from small-pox. It is the fashion now to recommend a re-vaccination, so as to be on the safe side, and to repeat it every few years. Dr. Carnochan, one of our most eminent surgeons, had his life recently threatened by the poison of a dead body coming in contact with a sore on his hand; the most infamous and loathsome of all diseases has often been communicated to healthy men by the matter of it coming in contact with a scratch on a delicate surface, as the lip, eye, or nostril; not only will these diseases be communicated by the vaccine matter taken from their arms, but vaccine matter taken from the arms of their children, whose blood is tainted from the father's, will communicate that same poison to a healthy person, and yet the child may look so healthy that it would seem unreasonable to fear any thing. Our conviction is, that if a vaccination has taken once, it is better to run the risk of variloid (modified small-pox), than to risk impregnation of disgusting disease by a repetition.

Increasing wealth has always induced increasing licentiousness, and very few of our young men in cities and large towns have escaped the diseases incident to licentiousness; these diseases are never eradicated wholly, and their children are tainted necessarily, which taint will be communicated to others, who are vaccinated from their arms. Under these circumstances, every family physician ought to be required to have matter on hand direct from the cow, or from the child of parents who have been "farming it" for twenty years, far off in the country; otherwise, our infants will be made scrofulous by our own acts, to die piece-meal, by lingering and painful maladies, entitled "obscure." Small-pox is more easily managed than formerly, and leaves the system less exposed to all diseases; and it may soon become a question whether it be not better to have small-pox than to risk by vaccination, especially by re-vaccination, the dangers just referred to.

